# Lack of bidirectional association between C-reactive protein and depressive symptoms in middle-aged and older adults: Results from a nationally representative prospective cohort study

**DOI:** 10.3389/fpsyg.2023.1095150

**Published:** 2023-02-13

**Authors:** Xiaohui Li, You Nie, Biru Chang

**Affiliations:** ^1^State Key Laboratory of Experimental Hematology, Fifth Medical Center of Chinese PLA General Hospital, Beijing, China; ^2^Capital Medical University, Beijing, China; ^3^School of Preschool Education, Xi’an University, Xi’an, China; ^4^Department of Psychology, Research Institute for International and Comparative Education, Shanghai Normal University, Shanghai, China

**Keywords:** C-reactive protein, depressive symptoms, longitudinal study, cross-lag model, national representative data set

## Abstract

Depression is associated with low quality of life and increased health burdens for middle-aged and older adults in resource-limited settings. Although inflammation plays an etiological role in the development and progression of depression, the directionality of the inflammation-depression relationship is unclear, especially in non-Western populations. To examine this relationship among community-dwelling Chinese middle-aged and older adults, we obtained data from the 2011, 2013, and 2015 China Health and Retirement Longitudinal Study (CHARLS). The participants were aged 45 years or above at baseline in 2011 and completed the follow-up survey in 2013 and 2015. Depressive symptoms were measured using the 10-item Center for Epidemiologic Studies Depression Scale (CESD-10), and the C-reactive protein (CRP) level was used to measure individual inflammation levels. Cross-lagged regression analyses examined the inflammation-depression relationship. Cross-group analyses were performed to test for model invariance across the sexes. Pearson’s correlations revealed no concurrent correlations between depression and CRP for both 2011 and 2015 (*p*s > 0.05, ranging 0.07–0.36) studies. Cross-lagged regression path analyses revealed that the paths from baseline CRP to depression in 2013 (*ß_std_* = −0.01, *p* = 0.80), from baseline CRP to depression in 2015 (*ß_std_* = 0.02, *p* = 0.47), from baseline depression to CRP in 2015 (*ß_std_* = −0.02, *p* = 0.40), and from depression at 2013 to CRP in 2015 (*ß_std_* = 0.03, *p* = 0.31) were not statistically significant. Additionally, the autoregressive model did not vary across the sexes (△*χ*^2^ = 78.75, *df* = 54, *p* = 0.02, △ comparative fit index (CFI) <0.01). We failed to find a bidirectional association between the CRP levels and depressive symptoms in our sample.

## 1. Introduction

Depression, associated with low quality of life and increased health burdens, is a disabling and prevailing mental health issue (Sivertsen et al., 2015; Wilkinson et al., 2018), with more than 264 million people affected worldwide (James et al., 2018). Middle-aged and older adults living in resource-limited settings (e.g., China) are disproportionally affected by depression and physical disability (James et al., 2018). Although several theories have been used to interpret the potential association between depression and physical health, the inflammation process has drawn considerable attention from psychologists and psychiatrists (Raison et al., 2006; Miller et al., 2009; Slavich and Irwin, 2014). Several theories have interpreted the bidirectional association of depression and inflammation, and the macrophage theory of depression hypothesizes that the onset and progression of depression are associated with the amount of pro-inflammatory cytokines secreted by the macrophages (Smith, 1991). Inflammation plays an etiologic role in the development and progression of depression, probably by altering the neurotransmitter metabolism or hypothalamic–pituitary–adrenal (HPA) axis function (Raison et al., 2006). HPA hyperactivity and the autonomic nervous system have also been used to explain how depression affects the inflammation processes (Kop and Gottdiener, 2005). Previous meta-analytic studies reported that older participants with depression demonstrated a high level of dysregulation of HPA axis activity through several mechanisms, including physical illnesses (e.g., diabetes, cardiovascular disease, and autoimmune diseases), alterations in the central nervous system (CNS), and immune-endocrinological alterations. These proposed biological mechanisms suggest a bidirectional path of the depression-inflammation association. Additionally, inflammation may trigger all-out-effort sickness behaviors, many of which may be associated with depressive symptoms (Hart, 1988). At the same time, depression could be prospectively associated with inflammation *via* behavioral changes (such as exercise, diet, and substance use) that are associated with inflammation, simply increasing experienced stress (which might increase inflammation).

One of the commonly and frequently studied inflammation biomarkers is C-reactive protein (CRP). It is an acute phase plasma protein with a short plasma half-life and a relatively robust and reliable response to inflammation, making CRP an ideal marker of inflammation. The high-sensitivity test for CRP, called low-reactive protein (LRP, L-CRP, or hs-CRP), more accurately measures very low levels of CRP, and static sampling of CRP has been widely used in clinical studies to predict diseases, such as cancer and depression (Coventry et al., 2009; Felger et al., 2020; Nehring et al., 2022). In the development, progression and treatment of depression, an increasing number of researchers have focused on the association between CRP levels and depressive symptoms [including major depressive disorder (MDD) and treatment-resistant depression (TRD)] (Orsolini et al., 2022). Several meta-analyses have explored the association between CRP and depression in participants with either major or probable depression and found significant but small effect sizes (Howren et al., 2009; Valkanova et al., 2013; Haapakoski et al., 2015; Horn et al., 2018). More importantly, when considering the study quality of the original studies, the effect size was strikingly attenuated and nonsignificant in the most recent meta-analysis (Horn et al., 2018). Whether this association exists in studies conducted among middle-aged and older adults is highly debatable (Penninx et al., 2003; Almeida et al., 2007; Bremmer et al., 2008; Pan et al., 2008; Stewart et al., 2008; Surtees et al., 2008; Almeida et al., 2009; Elovainio et al., 2009; Milaneschi et al., 2009; Su et al., 2009; Forti et al., 2010; Slopen et al., 2010; Bjerkeset et al., 2011; Häfner et al., 2011; Morris et al., 2011; Baune et al., 2012; Smagula et al., 2014; Zoga et al., 2014; Song et al., 2015; Tully et al., 2015; de Menezes et al., 2017). Although most studies show a significant positive relationship in either depressed or nondepressed older adults, some well-conducted studies failed to replicate these results (Almeida et al., 2009; Su et al., 2009; Forti et al., 2010; Morris et al., 2011; Baune et al., 2012; Häfner et al., 2012; de Menezes et al., 2017). Most studies lack (1) a random sampling method, which may result in potential sampling bias; (2) longitudinal study design, which is needed for assessing the temporal relationship and bi-direction; (3) statistical strictness in choosing confounders, and (4) detailed report of data cleaning strategies.

The present study investigated the relationship between hs-CRP levels and depression scores in middle-aged and older adults using longitudinal analysis in a nationally representative cohort. We used available data from the China Health and Retirement Longitudinal Study (CHARLS), which was designed to describe and prospectively delineate the social-, economic-and functional status of middle-aged and older adults. The CHARLS database provides a unique opportunity to examine the temporal and bidirectional relationship between depression and hs-CRP in middle-aged and older adults over 5 years while controlling for various socio-economic, anthropometric, physiologic, and psychosocial factors.

## 2. Materials and methods

### 2.1. Sample recruitment and dataset

This study’s data came from three waves of the China Health and Retirement Longitudinal Study (CHARLS 2011, 2013, and 2015), including depression and CRP assessments. This nationally representative sample recruited participants aged 45 years or above from 450 communities and 150 counties/districts, including 17,705 respondents from 10,257 households. A baseline survey was conducted between June 2011 and March 2012. Follow-up interviews were conducted between 2013 and 2015. The overall response rate for this survey at the baseline was 80.5%. More detailed information on CHARLS has been published previously (Zhao et al., 2014) and can also be found at.^1^ At baseline and follow-up assessment, 5,092 participants had no missing data on depression and CRP levels. Supplementary Figure S1 presents the detailed participant selection procedure.

### 2.2. Assessment of depression

The 10-item Center for Epidemiologic Studies Depression Scale (CESD-10) was used to measure depression levels. Radlo developed the original CESD, a reliable and valid screening tool in many previous community-based studies of the elderly (Radloff, 1977; Beekman et al., 1995, 1997; Lewinsohn et al., 1997). Researchers have developed various shorter forms of the CESD to simplify this scale and improve its sensitivity (Kohout et al., 1993). More recently, a 10-item short version of the CESD (CESD-10), derived from an analysis of item-total correlation (Andresen et al., 1994), proved to be comparably accurate to the original CES-D and is a reliable and valid depression assessment tool for older participants in different countries (Boey, 1999; Li et al., 2018, 2019). Each item was scored from 0 to 3, with a total CESD-10 score ranging from 0 to 30 (Li et al., 2018). In the present study, the Cronbach’s Alphas for CESD-10 ranged between 0.80 and 0.81 in 2011, 2013, and 2015.

### 2.3. Assessment of CRP

Based on the standard protocol, medically trained staff from the Chinese Center for Disease Control and Prevention (China CDC) collected venous blood at baseline and follow-up visits, mostly at centralized locations. All participants were asked to fast overnight, and over 92% of the participants who gave blood did fast. CRP levels were measured using an immunoturbidimetric assay at the Youanmen Center for the Clinical Laboratory of Capital Medical University. The within-assay coefficient was <1.3%, and the between-assay coefficient was <5.7%, with a detection limit of 0.1–20 mg/l. Additionally, the assay kits and methods for CRP assessment were kept constant at all the time points.

### 2.4. Control variables

According to previous suggestions (Horn et al., 2018), several additional variables of socio-demographic characteristics, health and behavior, and current medical treatment relevant to the relationship between depression and CRP were measured at the baseline visits. These variables included: age (birth year), sex (1 = male, 2 = female), education level (1 = below high school, 2 = high school or above), hukou status (1 = agricultural, 2 = non-agricultural hukou), marital status (1 = married, 2 = separated, divorced and widowed, 3 = never married), BMI (calculated as weight in kilograms divided by height in meters squared), smoking status (0 = nonsmoker, 1 = current smoker), alcohol status (0 = nondrinker, 1 = current drinker), and current medical treatment (including dyslipidemia, hypertension, and diabetes). All of these variables were coded as a binary variable (0 = no treatment, 1 = treatment). Demographic characteristics and behavioral and health indicator parameters were all influential factors for confounding variables. Table 1 presents the detailed descriptive statistics for these variables.

**Table 1:** Characteristics of participants (*N* = 5,092).

Variables	2011	2013	2015
	M (SD)/*n* (%)	M (SD)/*n* (%)	M (SD)/*n* (%)
**Demographic characteristic variables**			
Age (years) %	58.79 (8.49)	60.79 (8.49)	62.79 (8.49)
**Sex**			
Male	2,367 (46.5)	2,367 (46.5)	2,367 (46.5)
Female	2,721 (53.5)	2,721 (53.5)	2,721 (53.5)
**Hukou**			
Agricultural	4,183 (82.2)	4,183 (82.2)	4,183 (82.2)
Non-agricultural	907 (17.8)	907 (17.8)	907 (17.8)
**Education level**			
Below high school	4,536 (89.1)	4,536 (89.1)	4,536 (89.1)
High school or above	555 (10.9)	555 (10.9)	555 (10.9)
**Marital status**			
Married	4,628 (90.9)	2,367 (46.5)	4,466 (87.7)
Separated, divorced and widowed	433 (8.5)	510 (10.0)	594 (11.7)
Never married	31 (0.6)	35 (0.7)	32 (0.6)
**Health and behavior variables**			
BMI %	23.81 (3.94)	24.09 (3.93)	24.07 (4.03)
Smoking status			
No	3,557 (69.9)	3,557 (69.9)	3,706 (72.8)
Yes	1,534 (30.1)	1,534 (30.1)	1,384 (27.2)
**Alcohol status**			
No	3,381 (66.4)	3,381 (66.4)	3,333 (65.5)
Yes	1711 (33.6)	1711 (33.6)	1758 (34.5)
**Current medical treatment**			
Anti-dyslipidemia			
No	4,796 (94.2)	4,732 (92.9)	4,633 (91.0)
Yes	296 (5.8)	360 (7.1)	459 (9.0)
**Anti-hypertension**			
No	4,116 (80.8)	3,891 (76.4)	3,734 (73.3)
Yes	976 (19.2)	1,201 (23.6)	1,358 (26.7)
**Anti-diabetes**			
No	4,895 (96.1)	4,813 (94.5)	4,775 (93.8)
Yes	197 (3.9)	279 (5.5)	317 (6.2)
**Preliminary variables**			
CRP *^%^*	1.50 (1.55)	-	1.93 (1.76)
Depression *^%^*	8.26 (6.24)	8.13 (6.01)	8.14 (6.36)

Values are M (SD) = means (standard deviations) for continuous variables with % above and percentages (%) for categorical variables. BMI, body-mass index; CRP, C-reactive protein; -, no data.

### 2.5. Statistical analyses

We adopted SPSS 25.0^2^ to analyze the normality, missing effects by comparing the analytic sample and the total sample, the analytic and complete T1 samples, and the analytic and lost samples; correlations among control variables, CRP, and depression; and the between-group differences in CRP and depression and their effect size. Additionally, we adopted the Mplus 7.40 to conduct auto-regressive and cross-lag modeling (Muthén and Muthén, 1998–2012).

Sample difference comparisons were conducted for socio-demographic characteristics, health and behaviors, current medical treatment, CRPs and depression at both time points. With reference to previous reports (Stewart et al., 2009; Horn et al., 2018), we constructed four models to examine the longitudinal relations between CRP and depression: (1) the baseline model, namely the autoregressive model, reporting synchronous relations and all stability coefficients; (2) two unidirectional models, namely the depression main-effect model and CRP main-effects model; and (3) the reciprocal model that hypothesized depression and CRP could affect each other at different time points.

Following Byrne’s recommendations (Hayes-Ryan and Byrne, 2012), we adopted multiple indices to evaluate the goodness of fit for cross-lagged models: the Comparative Fit Index (CFI) with values higher than 0.90 indicative of an acceptable fit; the Root Mean Square Error of Approximation (RMSEA) and the Standardized Root Mean Square Residual (SRMR), with values below 0.08 indicative of an acceptable fit. Additionally, to examine the between-group differences between potential models (female vs. male), we followed Cheung and Rensvold’s suggestion that the changes in CFI (△CFI) less than 0.01 indicate invariance between the different models (Cheung and Rensvold, 2002).

Finally, the effect sizes for *t*-tests were computed with Cohen’s *d* using 0.2, 0.5, and 0.8 as lower bounds for small, medium, and large effects, respectively; the effect sizes for *χ^2^* test were computed with Cramer’s V and the effect sizes for *F* tests were computed with *η^2^* using 0.01, 0.059, and 0.138 as lower bounds for small, medium, and large effects (Cohen, 1988; Ehrminger et al., 2019). If the size of the missing effects was small, we considered our dataset to be a nonbiased sample.

## 3. Results

### 3.1. Preliminary analyses

First, the normal distribution tests demonstrated that the values of skewness and kurtosis for depression across 2011, 2013, and 2015 ranged from 0.84 to 0.97 and from 0.17 to 0.79, respectively; the values of skewness and kurtosis for CRP across 2011 and 2015 ranged from 1.96 to 2.34 and from 4.29 to 6.25, respectively (detailed information in Supplementary Table S1).

Second, sample difference comparisons indicated (1) no significant difference between the sample with no missing data in 2011 and the total sample compliance with inclusion criteria; (2) no significant differences between the sample with no missing data in 2011 and the analytic sample but age; and (3) no significant difference between the analytic sample and the lost sample but age, marital status, and anti-hypertension. These results indicate that older participants were inclined to be lost. Supplementary Tables S2–S4 provide the detailed information.

Third, regarding the effects of control variables on CRP and depression (Table 2), we found that most control variables were significantly correlated with depression and CRP in 2011 and 2015 (*p*s < 0.05, ranging from 0.00 to 0.04).

**Table 2:** The effects of categorical control variables on depression and CRP.

Control variables	Categories	CRP at 2011	Depression at 2011	Depression at 2013	CRP at 2015	Depression at 2015
		M (SD)	M (SD)	M (SD)	M (SD)	M (SD)
Sex	Male	**1.56 (1.61)**	**7.25 (5.79)**	**6.91 (5.17)**	1.95 (1.81)	**7.00 (5.87)**
	Female	**1.46 (1.49)**	**9.14 (6.49)**	**8.67 (6.02)**	1.92 (1.71)	**9.13 (6.61)**
Hukou status	Agricultural hukou	**1.47 (1.54)**	**8.62 (6.35)**	**8.16 (5.81)**	1.92 (1.74)	**8.54 (6.45)**
	Non-agricultural hukou	**1.64 (1.60)**	**6.63 (5.44)**	**6.45 (4.96)**	2.02 (1.85)	**6.31 (5.60)**
Education level	Below high school	1.50 (1.54)	**8.54 (6.32)**	**8.05 (5.76)**	**1.95 (1.77)**	**8.42 (6.43)**
	High school or above	1.57 (1.62)	**5.96 (4.96)**	**6.18 (4.96)**	**1.84 (1.69)**	**5.83 (5.21)**
Marital status	Married	1.49 (1.53)	**8.08 (6.16)**	**5.61 (0.08)**	1.92 (1.75)	**6.22 (0.09)**
	Separated, divorced and widowed	1.68 (1.69)	**9.93 (6.72)**	**6.29 (0.28)**	1.96 (1.80)	**7.14 (0.29)**
	Never married	1.59 (1.79)	**11.84 (6.67)**	**6.78 (1.15)**	2.67 (2.48)	**7.15 (1.26)**
Smoking status	Nonsmoker	1.48 (1.52)	**8.53 (6.31)**	**8.08 (5.81)**	1.94 (1.74)	**8.40 (6.44)**
	Smoker	1.55 (1.63)	**7.63 (6.02)**	**7.33 (5.43)**	1.93 (1.81)	**7.44 (6.10)**
Alcohol status	Nondrinker	1.51 (1.53)	**8.61 (6.32)**	**8.26 (5.81)**	1.97 (1.77)	**8.56 (6.46)**
	Drinker	1.49 (1.59)	**7.58 (6.03)**	**7.04 (5.42)**	1.87 (1.74)	**7.33 (6.11)**
Anti-dyslipidemia	No	**1.49 (1.54)**	**8.20 (6.22)**	**7.73 (5.65)**	**1.90 (1.75)**	**7.98 (6.27)**
	Yes	**1.80 (1.63)**	**9.29 (6.52)**	**9.48 (6.22)**	**2.31 (1.79)**	**9.74 (7.06)**
Anti-hypertension	No	**1.43 (1.50)**	**8.08 (6.21)**	**7.65 (5.60)**	**1.81 (1.71)**	**7.85 (6.22)**
	Yes	**1.84 (1.69)**	**9.02 (6.30)**	**8.50 (6.00)**	**2.27 (1.84)**	**8.94 (6.68)**
Anti-diabetes	No	**1.49 (1.54)**	8.24 (6.24)	**7.80 (5.71)**	**1.90 (1.74)**	**8.07 (6.35)**
	Yes	**1.76 (1.70)**	8.85 (6.32)	**8.68 (5.64)**	**2.38 (1.98)**	**9.15 (6.58)**

Values are M (SD) = means (standard deviations). BMI, body-mass index; CRP, C-reactive protein. Significant differences were written in bold.

Fourth, regarding the concurrent correlation (Table 3), we found that depression was not concurrently associated with CRP levels in 2011 (*r* = −0.01, *p =* 0.36). Similarly, four-year later depression was not related to four-year later CRP (*r* = 0.03, *p =* 0.07). Age and BMI were positively associated with CRP and depression in 2011, BMI was positively associated with depression in 2013, age and BMI were positively associated with CRP in 2015, and BMI was negatively associated with depression in 2015.

**Table 3:** Correlation analyses between continuous control variables, depression and CRP.

	Variables at 2011				Variables at 2013			Variables at 2015			
	Age	BMI	CRP	Depression	Age	BMI	CRP	Age	BMI	CRP	Depression
**Variables at 2011**											
Age	1										
BMI	−0.16**	1									
CRP	0.09**	0.02**	1								
Depression	0.05**	−0.09**	−0.01	1							
**Variables at 2013**											
Age	1.00**	−0.16**	0.09**	0.05**	1						
BMI	−0.18**	0.76**	0.13**	−0.05**	−0.18**	1					
Depression	−0.01	−0.06**	−0.01	0.51**	−0.01	0.05**	1				
**Variables at 2015**											
Age	1.00**	−0.16**	0.09**	0.05**	1.00**	−0.18**	−0.01	1			
BMI	−0.17**	0.74**	0.12**	−0.07**	−0.17**	0.77**	−0.06**	−0.17**	1		
CRP	0.06**	0.15**	0.31**	0.01	0.06**	0.18**	0.01	0.06**	0.18**	1	
Depression	0.01	−0.05**	−0.01	0.49**	0.01	−0.04*	0.55**	0.01	−0.05**	0.03	1

CRP, C-reactive protein. *p < 0.05. **p < 0.01.

### 3.2. Cross-lagged analyses

**Figure 1:**
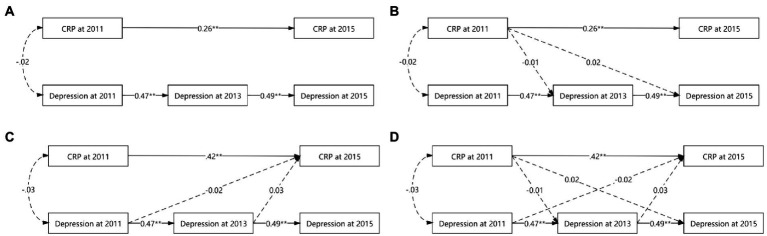
Various cross-lagged models’ figures. **(A)** for autoregressive model. **(B)** For CRP main-effect model. **(C)** For depression main-effect model. **(D)** For reciprocal model. To simplify the figure, the effects of the control variables used and the errors were omitted. While solid lines indicated significant correlation, dashed lines were not. *p < 0.05. **p < 0.01.

Although the autoregressive, CRP main-effect, depression main-effect, and reciprocal models yielded a good fit to the data across all fit indices, the other three models did not significantly differ from the autoregressive model (△*χ*^2^s > 0.00, *ps* > 0.05, △CFIs<0.01). Table 4 and Figure 1 provide more detailed results of these analyses.

**Table 4:** Fit indices of the various cross-lagged models.

Model description	*χ^2^*	df	*χ^2^*/df	CFI	SRMR	RMSEA	[90% CI]	Δ*χ^2^*	ΔCFI
Model 1: Autoregressive model	435.66	290	1.50	0.90	0.05	0.013	[0.012, 0.014]	-	-
**Model 2: Unidirection models**									
Model 2a: CRP main-effect model	435.33	288	1.50	0.90	0.05	0.017	[0.016, 0.018]	−0.33	0.00
Model 2b: Depression main-effect model	435.70	288	1.51	0.90	0.05	0.017	[0.016, 0.018]	0.04	0.00
Model 3: Reciprocal model	435.36	286	1.52	0.90	0.05	0.019	[0.017, 0.022]	−0.30	0.00

CRP, C-reactive protein; CFI, the comparative fit index; RMSEA, the root mean square error of approximation; SRMR, the standardized root mean square residual.

In the autoregressive model, baseline CRP was positively associated with CRP in 2015 (*ß_std_* = 0.26, *p* < 0.01), baseline depression prospectively predict depression in 2013 (*ß_std_* = 0.47, *p* < 0.01), and depression in 2013 prospectively predicted depression in 2015 (*ß_std_* = 0.49, *p* < 0.01).

Based on the autoregressive model, the path from baseline CRP to depression in 2013 (*ß_std_* = −0.01, *p* = 0.81) and the path from baseline CRP to depression in 2015 (*ß_std_* = 0.02, *p* = 0.47) were not significant in the CRP main-effect model, suggesting that baseline CRP could not prospectively predict depression at further time points after controlling all synchronous relations and all stability coefficients.

Based on the autoregressive model, the path from baseline depression to CRP in 2015 (*ß_std_* = −0.02, *p* = 0.41) and the path from depression in 2013 to CRP in 2015 (*ß_std_* = 0.03, *p* = 0.30) were not significant in the depression main-effect model, suggesting that depression could not prospectively predict CRP at further time points after controlling all synchronous relations and all stability coefficients.

Based on the autoregressive model, the paths from baseline CRP to depression in 2013 (*ß_std_* = −0.01, *p* = 0.80), from baseline CRP to depression in 2015 (*ß_std_* = 0.02, *p* = 0.47) were not significant in the CRP main-effect model, from baseline depression to CRP in 2015 (*ß_std_* = −0.02, *p* = 0.40), and from depression in 2013 to CRP in 2015 (*ß_std_* = 0.03, *p* = 0.31) were not significant in the CRP-depression reciprocal model, suggesting that CRP and depression could not prospectively predict each other at further time points after controlling all synchronous relations and all stability coefficients.

Cross-group analyses were also conducted to determine whether sex moderated any of the observed relationships. Again, the difference tests indicated that the autoregressive model did not vary significantly across the sexes (△*χ^2^* = 78.75, *df* = 54, *p* = 0.02, △CFI < 0.01).

## 4. Discussion

This study investigated whether the inflammatory marker, CRP, could prospectively predict symptoms of depression in middle-aged and older adults using a prospective design of community and rural dwellings, accounting for a range of potential confounders. CRP was not significantly associated with depression both cross-sectionally and longitudinally in the largest sample. Consistent with the results of previous studies in some Asian regions, such as Islamabad, there was a lack of association between CRP and depression influenced by racial/ethnic, genetic, and environmental factors, and population-based assessments of associations between physiological processes or social integration should consider these variables (Chapman and Santos-Lozada, 2020; Zavos et al., 2022). Contrary to other reports, we did not find this association with gender modification either (Danner et al., 2003; Morris et al., 2011; Vogelzangs et al., 2012; Song et al., 2015; Köhler-Forsberg et al., 2017). In fact, it is unclear from existing studies whether CRP levels directly contribute to the onset and progression of depression.

Notably, BMI was negatively associated with depression in 2015 in our study, consistent with previous studies reported in Korea and China, and both studies demonstrated a negative correlation between BMI and depressive symptoms (Qin et al., 2017; Lee et al., 2019). This is contrary to the majority of findings from Western studies that reported a significant association between clinical overweighted or obesity and depression (Luppino et al., 2010; Daly, 2013; Preiss et al., 2013). Similarly, studies on other Asian populations have reported that being overweight or obese prevents depression. As previously reported, overweight individuals in Hong Kong and Bangladesh are less likely to have depressive symptoms, and obesity is an independent protective factor for depressive symptoms (Li et al., 2004). This counterintuitive epidemiological finding appears to be consistent with the “obesity paradox” theory, which reflects the relationship between obesity and reduced mortality compared with normal weight (Adams et al., 2006). However, the CESD-10 was used in the study to primarily reflect an individual’s experience of depression, and abnormalities in BMI (underweight and overweight) may affect a person’s body satisfaction and self-esteem, which in turn may affect the test results (Richard et al., 2016).

However, our study found no significant association between CRP level and depression both cross-sectionally and longitudinally. This is in contrast to most findings from Western studies, which reported a significantly higher prevalence of MDD in individuals with high CRP levels, and this correlation appears to be more prominent in younger adults than in older patients (Jung and Kang, 2019; Milaneschi et al., 2021; Orsolini et al., 2022). The cause of elevated CRP may be another potential mechanism of action for neurovascular injury induced by dysregulation of peripheral myeloid cells, pro-inflammatory cytokines and complement pathways (Menard et al., 2017; Horn et al., 2018). Additionally, elevated CRP levels and pro-inflammatory activity can drive inflammation through microglia and astrocyte activation (McKim et al., 2018; Wesselingh et al., 2019).

Our results lend partial support to studies on middle-aged and older adults that found no association between elevated CRP and higher odds of depression before and after adjusting for demographic, socio-economic, lifestyle, and prior medical history variables (Pan et al., 2008; Stewart et al., 2008, 2009; Song et al., 2015; de Menezes et al., 2017). Possible interpretations can be made based on two aspects: sampling methods and depression types. Some studies adopted a convenience sample, which may have caused selection bias. Our study adopted a four-stage random sampling method across China, minimizing the potential bias and magnifying the sample representativeness of older Chinese participants. Our results partially replicated studies conducted in some East Asian populations, who share similar lifestyles, such as a sample of 3,289 community residents from Beijing and Shanghai (Pan et al., 2008) and a sample of 569 from a rural community in Korea (Song et al., 2015). However, two studies that adopted a random sampling method, were conducted in Whites, and found a unidirectional association, which suggests that race may play a role in this association (Gimeno et al., 2009; Zalli et al., 2016). Owing to the highly mixed results in this field, ethnic and racial differences should also be considered in future studies. Third, CRP-depression may only exist in people living with atypical depression rather than melancholic depression (Glaus et al., 2014; Lamers et al., 2016), which reflects that depression subtypes may have unique but different biological pathways. Further studies are needed to verify the neurobiological pathways of the CRP-depression association in different subtypes.

The strengths and limitations of this study should be mentioned. The major power is the strictness of the study design (random sampling method, prospective cohort, and large sample size), which supports high internal and external validity. However, this study had several limitations. First, we did not collect other pro-inflammatory biomarkers, like interleukin-6 (IL-6), which might be longitudinally associated with future depression status (Stewart et al., 2009). Second, the CESD-10 is not a commonly used assessment of depression in previous reports, limiting the comparability of our results to those of other studies. Third, CRP was collected in two waves, which limits our ability to assess the dynamic interaction between CRP and depression. Fourth, the interval between participant visits is too long, which may cause dampening effects of potential associations or stronger effects due to long-term interactions between variables.

## 5. Conclusion

In summary, we failed to find a bidirectional association between CRP levels and depressive symptoms in well-represented middle-aged and older Chinese adults. Additionally, we did not observe any sex-related modifications. Future studies should validate the role of multifaceted pro-inflammatory biomarkers in predicting depression and should also consider racial/ethnic, genetic, and environmental factors for the lack of association between CRP and depression and the use of commonly used depression scales and smaller time intervals to make the results comparable.

## Data availability statement

The datasets presented in this study can be found in online repositories. The names of the repository/repositories and accession number(s) can be found in the article/Supplementary material.

## Ethics statement

Written informed consent was obtained from the individual(s) for the publication of any potentially identifiable images or data included in this article.

## Author contributions

BC formulated the research questions, designed the study, supervised the data cleaning. XL wrote the paper. BC and YN carried out data consolidation, cleaning, and preliminary analysis. XL, BC, and YN were responsible for principal analysis and result interpretation. XL and BC were responsible for the revision of the manuscript. All authors contributed to the article and approved the submitted version.

## Conflict of interest

The authors declare that the research was conducted in the absence of any commercial or financial relationships that could be construed as a potential conflict of interest.

## Publisher’s note

All claims expressed in this article are solely those of the authors and do not necessarily represent those of their affiliated organizations, or those of the publisher, the editors and the reviewers. Any product that may be evaluated in this article, or claim that may be made by its manufacturer, is not guaranteed or endorsed by the publisher.

## Footnotes

^1^http://charls.pku.edu.cn/

^2^www.ibm.com/products/spss-statistics
