# Supplementary material for: Lack of bidirectional association between C-reactive protein and depressive symptoms in middle-aged and older adults: Results from a nationally representative prospective cohort study
Source: Front Psychol. 2023 Feb 13;14:1095150. doi: 10.3389/fpsyg.2023.1095150 (PMC9969160; doi:10.3389/fpsyg.2023.1095150)
Supplement: Supplementary file 1 [file Data_Sheet_1.docx]

Supplementary Material

# Supplementary Figures and Tables

## Supplementary Figures


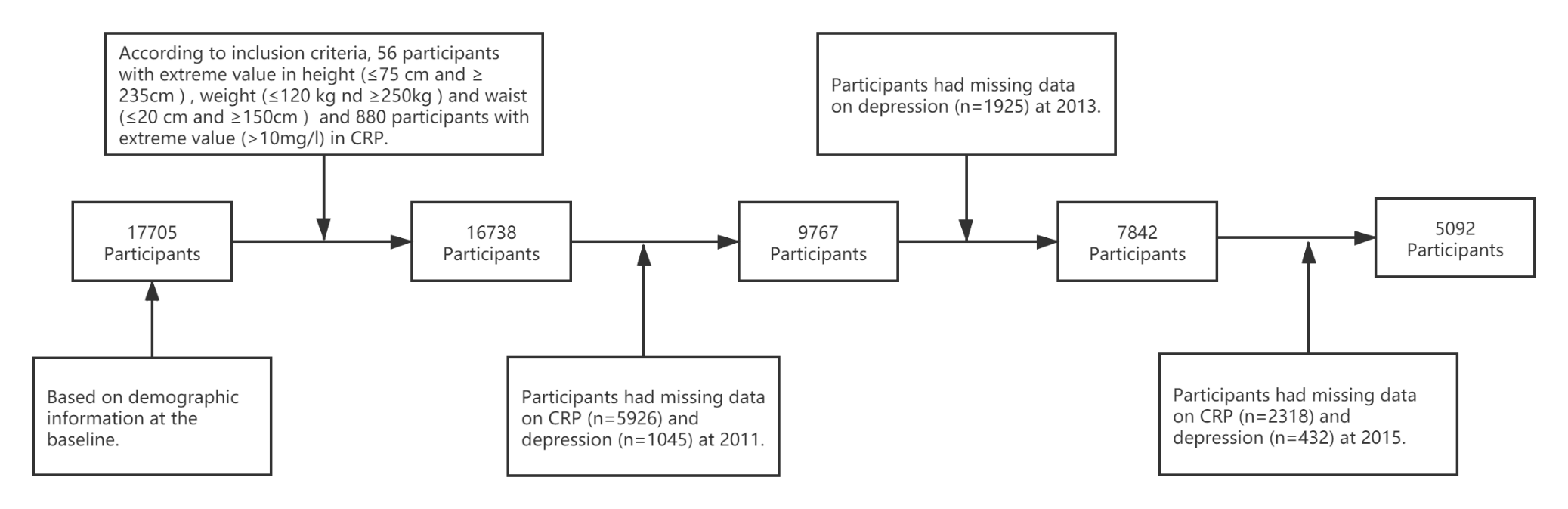


**Supplementary Figure 1.** (S1 Figure) Selection procedure of depression and CRP raw data

## Supplementary Tables

**Supplementary Table 1** (S1 Table) The normal distribution test for continuous variables

| **Variables** | N | Minimum | Maximum | M (SD) | Skewness | Kurtosis |
| --- | --- | --- | --- | --- | --- | --- |
| **Variables at 2011** |  |  |  |  |  |  |
| Age (years) | 5083 | 32.00 | 94.00 | 58.79 (8.49) | 0.35 | -0.29 |
| BMI | 4607 | 12.97 | 71.29 | 23.81 (3.94) | 1.62 | 10.63 |
| CRP | 5092 | 0.01 | 9.97 | 1.5 (1.55) | 2.34 | 6.25 |
| Depression | 5092 | 0.00 | 30.00 | 8.26 (6.24) | 0.84 | 0.17 |
| **Variables at 2013** |  |  |  |  |  |  |
| Age (years) | 5083 | 34.00 | 96.00 | 60.79 (8.49) | 0.35 | -0.29 |
| BMI | 4260 | 13.20 | 103.41 | 24.09 (3.93) | 2.59 | 40.96 |
| Depression | 5092 | 0.00 | 30.00 | 7.85 (5.71) | 0.97 | 0.79 |
| **Variables at 2015** |  |  |  |  |  |  |
| Age (years) | 5083 | 36.00 | 98.00 | 62.79 (8.49) | 0.35 | -0.29 |
| BMI | 5024 | 13.43 | 71.43 | 24.07 (4.03) | 2.00 | 16.72 |
| CRP | 5092 | 0.10 | 10.00 | 1.93 (1.76) | 1.96 | 4.29 |
| Depression | 5092 | 0.00 | 30.00 | 8.14 (6.36) | 0.95 | 0.44 |

Notes. M (SD)=means (standard deviations). BMI=body-mass index. CRP=C-reactive protein.

**Supplementary Table 2** (S2 Table) Comparison between the total sample compliance with inclusion criteria (n=17738) and the sample with no missing data at 2011 (n=9767)

| **Variables** | | **Mean (SD) or distribution (%)** | | **Comparison** |  | **Effect size** |
| --- | --- | --- | --- | --- | --- | --- |
|  |  | **The total sample compliance with inclusion criteria** | **The sample with no missing data at 2011** | **Test (*t / χ2*)** | ***p*** | ***d / V*** |
| **Demographic characteristic variables at 2011** |  |  |  |  |  |  |
| Age (years) *^%^* |  | 59.88 (10.15) | 59.73 (9.56) | 1.23 | 0.22 | 0.02 |
| Sex | Male | 7960 (47.6) | 4484 (45.9) | 6.89 | 0.03 | 0.02 |
|  | Female | 8766 (52.4) | 5276 (54.1) |  |  |  |
| Hukou | Agricultural hukou | 12909 (77.3) | 7859 (80.5) | 37.77 | *p*≤0.001 | 0.04 |
|  | Non-agricultural hukou | 3799 (22.7) | 1906 (19.5) |  |  |  |
| Education level | Below high school | 14518 (87.0) | 8647 (88.5) | 8.02 | 0.02 | 0.02 |
|  | High school or above | 2170 (13.0) | 1106 (11.3) |  |  |  |
| Marital status | Married | 14597 (87.4) | 8647 (88.5) | 8.02 | 0.02 | 0.02 |
|  | Separated, divorced and widowed | 1966 (11.8) | 1046 (10.7) |  |  |  |
|  | Never married | 146 (0.9) | 74 (0.8) |  |  |  |
| **Health and behavior variables at 2011** |  |  |  |  |  |  |
| BMI *^%^* |  | 23.47 (3.95) | 23.57 (3.93) | 1.73 | 0.08 | 0.03 |
| Smoking status | No | 11425 (71.5) | 6886 (70.5) | 3.05 | 0.08 | 0.01 |
|  | Yes | 4525 (28.5) | 2878 (29.5) |  |  |  |
| Alcohol status | No | 11140 (67.2) | 6609 (67.7) | 0.73 | 0.40 | 0.01 |
|  | Yes | 5448 (32.8) | 3158 (32.3) |  |  |  |
| **Current medical treatment at 2011** |  |  |  |  |  |  |
| Anti-dyslipidemia | No | 15899 (95.0) | 9242 (94.6) | 1.66 | 0.20 | 0.01 |
|  | Yes | 839 (5.0) | 525 (5.4) |  |  |  |
| Anti-hypertension | No | 13577 (81.1) | 7837 (80.2) | 3.05 | 0.08 | 0.01 |
|  | Yes | 3161 (18.9) | 1930 (19.8) |  |  |  |
| Anti-diabetes | No | 16085 (96.1) | 9384 (96.1) | 0.01 | 0.94 | 0.00 |
|  | Yes | 653 (3.9) | 383 (3.9) |  |  |  |
| **Preliminary variables at 2011** |  |  |  |  |  |  |
| CRP *^%^* |  | 1.57 (1.66) | 1.58 (1.65) | 0.06 | 0.95 | 0.01 |
| Depression *^%^* |  | 8.35 (6.34) | 8.56 (6.40) | 2.43 | 0.02 | 0.03 |
| **Demographic characteristic variables at 2013** |  |  |  |  |  |  |
| Age (years) *^%^* |  | 61.88 (10.15) | 61.73 (9.56) | 1.23 | 0.22 | 0.02 |
| Sex | Male | 7960 (47.6) | 4484 (45.9) | 6.89 | 0.03 | 0.02 |
|  | Female | 8766 (52.4) | 5276 (54.1) |  |  |  |
| Hukou | Agricultural hukou | 12909 (77.3) | 7859 (80.5) | 37.77 | *p*≤0.001 | 0.04 |
|  | Non-agricultural hukou | 3799 (22.7) | 1906 (19.5) |  |  |  |
| Education level | Below high school | 14518 (87.0) | 8647 (88.5) | 8.02 | 0.02 | 0.02 |
|  | High school or above | 2170 (13.0) | 1106 (11.3) |  |  |  |
| Marital status | Married | 12372 (86.5) | 7789 (87.4) | 4.12 | 0.13 | 0.01 |
|  | Separated, divorced and widowed | 1804 (12.6) | 1047 (11.8) |  |  |  |
|  | Never married | 123 (0.9) | 71 (0.8) |  |  |  |
| **Health and behavior variables at 2013** |  |  |  |  |  |  |
| BMI *^%^* |  | 23.81 (3.89) | 23.87 (3.91) | 1.04 | 0.30 | 0.02 |
| Smoking status | No | 11425 (71.5) | 6886 (70.5) | 3.05 | 0.08 | 0.01 |
|  | Yes | 4525 (28.5) | 2878 (29.5) |  |  |  |
| Alcohol status | No | 11140 (67.2) | 6609 (67.7) | 0.73 | 0.40 | 0.01 |
|  | Yes | 5448 (32.8) | 3158 (32.3) |  |  |  |
| **Current medical treatment at 2013** |  |  |  |  |  |  |
| Anti-dyslipidemia | No | 15810 (94.5) | 9157 (93.8) | 5.55 | 0.02 | 0.01 |
|  | Yes | 928 (5.5) | 610 (6.2) |  |  |  |
| Anti-hypertension | No | 13521 (80.8) | 7667 (78.5) | 20.02 | *p*≤0.001 | 0.03 |
|  | Yes | 3217 (19.2) | 2100 (21.5) |  |  |  |
| Anti-diabetes | No | 15950 (95.3) | 9264 (94.9) | 2.60 | 0.11 | 0.01 |
|  | Yes | 788 (4.7) | 503 (5.1) |  |  |  |
| **Preliminary variables at 2013** |  |  |  |  |  |  |
| Depression *^%^* |  | 7.90 (5.79) | 7.95 (5.82) | 0.72 | 0.47 | 0.01 |
| **Demographic characteristic variables at 2015** |  |  |  |  |  |  |
| Age (years) *^%^* |  | 63.88 (10.15) | 63.73 (9.56) | 1.23 | 0.22 | 0.02 |
| Sex | Male | 7960 (47.6) | 4484 (45.9) | 6.89 | 0.03 | 0.02 |
|  | Female | 8766 (52.4) | 5276 (54.1) |  |  |  |
| Hukou | Agricultural hukou | 12909 (77.3) | 7859 (80.5) | 37.77 | *p*≤0.001 | 0.04 |
|  | Non-agricultural hukou | 3799 (22.7) | 1906 (19.5) |  |  |  |
| Education level | Below high school | 14518 (87.0) | 8647 (88.5) | 8.02 | 0.02 | 0.02 |
|  | High school or above | 2170 (13.0) | 1106 (11.3) |  |  |  |
| Marital status | Married | 11618 (86.1) | 7382 (85.5) | 0.90 | 0.64 | 0.01 |
|  | Separated, divorced and widowed | 1933 (14.1) | 1180 (13.7) |  |  |  |
|  | Never married | 113 (0.8) | 67 (0.8) |  |  |  |
| **Health and behavior variables at 2015** |  |  |  |  |  |  |
| BMI *^%^* |  | 24.01 (8.80) | 24.03 (8.78) | 0.40 | 0.69 | 0.04 |
| Smoking status | No | 9959 (72.8) | 6352 (73.7) | 2.51 | 0.11 | 0.01 |
|  | Yes | 3724 (27.2) | 2261 (26.3) |  |  |  |
| Alcohol status | No | 9097 (66.5) | 5812 (67.5) | 2.17 | 0.14 | 0.01 |
|  | Yes | 4579 (33.5) | 2802 (32.5) |  |  |  |
| **Current medical treatment at 2015** |  |  |  |  |  |  |
| Anti-dyslipidemia | No | 15636 (93.4) | 9032 (92.5) | 8.48 | *p*≤0.001 | 0.02 |
|  | Yes | 1102 (6.6) | 735 (7.5) |  |  |  |
| Anti-hypertension | No | 13260 (79.2) | 7476 (76.5) | 25.97 | *p*≤0.001 | 0.03 |
|  | Yes | 3478 (20.8) | 2291 (23.5) |  |  |  |
| Anti-diabetes | No | 15915 (95.1) | 9238 (94.6) | 3.18 | 0.08 | 0.01 |
|  | Yes | 823 (4.9) | 529 (5.4) |  |  |  |
| **Preliminary variables at 2015** |  |  |  |  |  |  |
| CRP *^%^* |  | 1.90 (1.73) | 1.93 (1.76) | 1.15 | 0.25 | 0.04 |
| Depression *^%^* |  | 8.18 (6.44) | 8.30 (6.47) | 1.29 | 0.20 | 0.02 |

Notes. Values are M (SD) =means (standard deviations) for continuous variables with *%* above and percentages (%) for categorical variables. BMI=body-mass index. CRP=C-reactive protein. V in effects sizes=Cramer’s V. *d* in effects sizes=Cohen’s *d*. Comparison and effect size were reported in absolute values. Significant differences were written in bold.

**Supplementary Table 3** (S3 Table) Comparison between the sample with no missing data at 2011 (n=9767) and the analytic sample (n=5092)

| **Variables** |  | **Mean (SD) or distribution (%)** | | **Comparison** | | **Effect size** |
| --- | --- | --- | --- | --- | --- | --- |
|  |  | **The sample with no missing data at 2011** | **The analytic sample** | **Test (*t / χ2*)** | ***p*** | ***d / V*** |
| **Demographic characteristic variables at 2011** |  |  |  |  |  |  |
| Age (years) *^%^* |  | **59.73 (9.56)** | **58.79 (8.49)** | **6.11** | ***p*≤0.001** | **0.10** |
| Sex | Male | 4484 (45.9) | 2367 (46.5) | 0.96 | 0.62 | 0.01 |
|  | Female | 5276 (54.1) | 2721 (53.5) |  |  |  |
| Hukou | Agricultural hukou | 7859 (80.5) | 4183 (82.2) | 6.30 | 0.01 | 0.02 |
|  | Non-agricultural hukou | 1906 (19.5) | 907 (17.8) |  |  |  |
| Education level | Below high school | 8647 (88.5) | 4536 (89.1) | 0.61 | 0.44 | 0.01 |
|  | High school or above | 1106 (11.3) | 555 (10.9) |  |  |  |
| Marital status | Married | 8647 (88.5) | 4628 (90.9) | 19.49 | *p*≤0.001 | 0.04 |
|  | Separated, divorced and widowed | 1046 (10.7) | 433 (8.5) |  |  |  |
|  | Never married | 74 (0.8) | 31 (0.6) |  |  |  |
| **Health and behavior variables at 2011** |  |  |  |  |  |  |
| BMI *^%^* |  | 23.57 (3.93) | 23.81 (3.94) | 3.37 | *p*≤0.001 | 0.05 |
| Smoking status | No | 6886 (70.5) | 3557 (69.9) | 0.69 | 0.41 | 0.01 |
|  | Yes | 2878 (29.5) | 1534 (30.1) |  |  |  |
| Alcohol status | No | 6609 (67.7) | 3381 (66.4) | 2.44 | 0.12 | 0.01 |
|  | Yes | 3158 (32.3) | 1711 (33.6) |  |  |  |
| **Current medical**  **treatment at 2011** |  |  |  |  |  |  |
| Anti-dyslipidemia | No | 9242 (94.6) | 4796 (94.2) | 1.23 | 0.27 | 0.01 |
|  | Yes | 525 (5.4) | 296 (5.8) |  |  |  |
| Anti-hypertension | No | 7837 (80.2) | 4116 (80.8) | 0.75 | 0.39 | 0.01 |
|  | Yes | 1930 (19.8) | 976 (19.2) |  |  |  |
| Anti-diabetes | No | 9384 (96.1) | 4895 (96.1) | 0.03 | 0.88 | 0.00 |
|  | Yes | 383 (3.9) | 197 (3.9) |  |  |  |
| **Preliminary variables at 2011** |  |  |  |  |  |  |
| CRP *^%^* |  | 1.58 (1.65) | 1.50 (1.55) | 2.61 | 0.01 | 0.04 |
| Depression *^%^* |  | 8.56 (6.40) | 8.26 (6.24) | 2.72 | 0.01 | 0.04 |
| **Demographic characteristic variables at 2013** |  |  |  |  |  |  |
| Age (years) *^%^* |  | **61.73 (9.56)** | **60.79 (8.49)** | **6.11** | ***p*≤0.001** | **0.10** |
| Sex | Male | 4484 (45.9) | 2367 (46.5) | 0.96 | 0.62 | 0.01 |
|  | Female | 5276 (54.1) | 2721 (53.5) |  |  |  |
| Hukou | Agricultural hukou | 7859 (80.5) | 4183 (82.2) | 6.30 | 0.01 | 0.02 |
|  | Non-agricultural hukou | 1906 (19.5) | 907 (17.8) |  |  |  |
| Education level | Below high school | 8647 (88.5) | 4536 (89.1) | 0.61 | 0.44 | 0.01 |
|  | High school or above | 1106 (11.3) | 555 (10.9) |  |  |  |
| Marital status | Married | 7789 (87.4) | 4547 (89.3) | 10.58 | 0.01 | 0.03 |
|  | Separated, divorced and widowed | 1047 (11.8) | 510 (10.0) |  |  |  |
|  | Never married | 71 (0.8) | 35 (0.7) |  |  |  |
| **Health and behavior variables at 2013** |  |  |  |  |  |  |
| BMI *^%^* |  | 23.87 (3.91) | 24.09 (3.93) | 2.86 | *p*≤0.001 | 0.04 |
| Smoking status | No | 6886 (70.5) | 3557 (69.9) | 0.69 | 0.41 | 0.01 |
|  | Yes | 2878 (29.5) | 1534 (30.1) |  |  |  |
| Alcohol status | No | 6609 (67.7) | 3381 (66.4) | 2.44 | 0.12 | 0.01 |
|  | Yes | 3158 (32.3) | 1711 (33.6) |  |  |  |
| **Current medical conditions at 2013** |  |  |  |  |  |  |
| Anti-dyslipidemia | No | 9157 (93.8) | 4732 (92.9) | 3.73 | 0.05 | 0.02 |
|  | Yes | 610 (6.2) | 360 (7.1) |  |  |  |
| Anti-hypertension | No | 7667 (78.5) | 3891 (76.4) | 8.42 | *p*≤0.001 | 0.02 |
|  | Yes | 2100 (21.5) | 1201 (23.6) |  |  |  |
| Anti-diabetes | No | 9264 (94.9) | 4813 (94.5) | 0.73 | 0.39 | 0.01 |
|  | Yes | 503 (5.1) | 279 (5.5) |  |  |  |
| **Preliminary variables at 2013** |  |  |  |  |  |  |
| Depression *^%^* |  | 7.95 (5.82) | 7.85 (5.71) | 0.95 | 0.34 | 0.02 |
| **Demographic characteristic variables at 2015** |  |  |  |  |  |  |
| Age (years) *^%^* |  | **63.73 (9.56)** | **62.79 (8.49)** | **6.11** | ***p*≤0.001** | **0.10** |
| Sex | Male | 4484 (45.9) | 2367 (46.5) | 0.96 | 0.62 | 0.01 |
|  | Female | 5276 (54.1) | 2721 (53.5) |  |  |  |
| Hukou | Agricultural hukou | 7859 (80.5) | 4183 (82.2) | 6.30 | 0.01 | 0.02 |
|  | Non-agricultural hukou | 1906 (19.5) | 907 (17.8) |  |  |  |
| Education level | Below high school | 8647 (88.5) | 4536 (89.1) | 0.61 | 0.44 | 0.01 |
|  | High school or above | 1106 (11.3) | 555 (10.9) |  |  |  |
| Marital status | Married | 7382 (85.5) | 4466 (87.7) | 12.70 | *p*≤0.001 | 0.03 |
|  | Separated, divorced and widowed | 1180 (13.7) | 594 (11.7) |  |  |  |
|  | Never married | 67 (0.8) | 32 (0.6) |  |  |  |
| **Health and behavior variables at 2015** |  |  |  |  |  |  |
| BMI *^%^* |  | 24.03 (8.78) | 24.07 (4.03) | 0.24 | 0.81 | 0.01 |
| Smoking status | No | 6352 (73.7) | 3706 (72.8) | 1.45 | 0.23 | 0.01 |
|  | Yes | 2261 (26.3) | 1384 (27.2) |  |  |  |
| Alcohol status | No | 5812 (67.5) | 3333 (65.5) | 5.78 | 0.02 | 0.02 |
|  | Yes | 2802 (32.5) | 1758 (34.5) |  |  |  |
| **Current medical conditions at 2015** |  |  |  |  |  |  |
| Anti-dyslipidemia | No | 9032 (92.5) | 4633 (91.0) | 10.04 | *p*≤0.001 | 0.03 |
|  | Yes | 735 (7.5) | 459 (9.0) |  |  |  |
| Anti-hypertension | No | 7476 (76.5) | 3734 (73.3) | 18.65 | *p*≤0.001 | 0.04 |
|  | Yes | 2291 (23.5) | 1358 (26.7) |  |  |  |
| Anti-diabetes | No | 9238 (94.6) | 4775 (93.8) | 4.08 | 0.04 | 0.02 |
|  | Yes | 529 (5.4) | 317 (6.2) |  |  |  |
| **Preliminary variables at 2015** |  |  |  |  |  |  |
| CRP *^%^* |  | 1.93 (1.76) | 1.93 (1.76) | 0.06 | 0.95 | 0.00 |
| Depression *^%^* |  | 8.30 (6.47) | 8.14 (6.36) | 1.43 | 0.15 | 0.02 |

Notes. Values are M (SD) =means (standard deviations) for continuous variables with *%* above and percentages (%) for categorical variables. BMI=body-mass index. CRP=C-reactive protein. V in effects sizes=Cramer’s V. *d* in effects sizes=Cohen’s *d*. Comparison and effect size were reported in absolute values. Significant differences were written in bold.

**Supplementary Table 4** (S4 Table) Comparison between the analytic sample (n=5092) and the lost sample (n=4685)

| **Variables** |  | **Mean (SD) or distribution (%)** | | **Comparison** |  | **Effect size** |  |
| --- | --- | --- | --- | --- | --- | --- | --- |
|  |  | **The analytic sample** | **The lost sample** | **Test (*t / χ2*)** | ***p*** | ***d / V*** |  |
| **Demographic characteristic variables at 2011** |  |  |  |  |  |  |  |
| Age (years) *^%^* |  | **58.79 (8.49)** | **60.75 (10.5)** | **10.08** | ***p*≤0.001** | **0.19** |  |
| Sex | Male | 2367 (46.5) | 2118 (45.3) | 1.31 | 0.24 | 0.01 |  |
|  | *F*emale | 2721 (53.5) | 2555 (54.7) |  |  |  |  |
| Hukou | Agricultural hukou | 4183 (82.2) | 3676 (78.6) | 19.55 | *p*≤0.001 | 0.04 |  |
|  | Non-agricultural hukou | 907 (17.8) | 999 (21.4) |  |  |  |  |
| Education level | Below high school | 4536 (89.1) | 4123 (88.2) | 1.91 | 0.17 | 0.01 |  |
|  | High school or above | 555 (10.9) | 551 (11.8) |  |  |  |  |
| Marital status | Married | 4628 (90.9) | 4019 (86.0) | 48.12 | *p*≤0.001 | 0.04 |  |
|  | Separated, divorced and widowed | 433 (8.5) | 613 (13.1) |  |  |  |  |
|  | Never married | 31 (0.6) | 43 (0.9) |  |  |  |  |
| **Health and behavior variables at 2011** |  |  |  |  |  |  |  |
| BMI *^%^* |  | 23.81 (3.94) | 23.68 (3.90) | 6.17 | *p*≤0.001 | 0.04 |  |
| Smoking status | No | 3557 (69.9) | 3329 (71.2) | 2.20 | 0.14 | 0.01 |  |
|  | Yes | 1534 (30.1) | 1344 (28.8) |  |  |  |  |
| Alcohol status | No | 3381 (66.4) | 3228 (69.0) | 7.82 | *p*≤0.001 | 0.03 |  |
|  | Yes | 1711 (33.6) | 1447 (31.0) |  |  |  |  |
| **Current medical treatment at 2011** |  |  |  |  |  |  |  |
| Anti-dyslipidemia | No | 4796 (94.2) | 4446 (95.1) | 4.01 | 0.05 | 0.02 |  |
|  | Yes | 296 (5.8) |  |  |  |  |  |
| Anti-hypertension | No | 4116 (80.8) | 3721 (79.6) | 2.36 | 0.12 | 0.02 |  |
|  | Yes | 976 (19.2) | 954 (20.4) |  |  |  |  |
| Anti-diabetes | No | 4895 (96.1) | 4489 (96.0) | 0.08 | 0.78 | 0.00 |  |
|  | Yes | 197 (3.9) | 186 (4.0) |  |  |  |  |
| **Preliminary variables at 2011** |  |  |  |  |  |  |  |
| CRP *^%^* |  | 1.50 (1.55) | 1.58 (1.75) | 4.45 | *p*≤0.001 | 0.04 |  |
| Depression *^%^* |  | 8.26 (6.24) | 8.55 (6.55) | 4.78 | *p*≤0.001 | 0.04 |  |
| **Demographic characteristic variables at 2013** |  |  |  |  |  |  |  |
| Age (years) *^%^* |  | **60.79 (8.49)** | **62.75 (10.5)** | **0.08** | ***p*≤0.001** | **0.19** |  |
| Sex | Male | 2367 (46.5) | 2118 (45.3) | 1.31 | 0.24 | 0.01 |  |
|  | Female | 2721 (53.5) | 2555 (54.7) |  |  |  |  |
| Hukou | Agricultural hukou | 4183 (82.2) | 3676 (78.6) | 19.55 | *p*≤0.001 | 0.04 |  |
|  | Non-agricultural hukou | 907 (17.8) | 999 (21.4) |  |  |  |  |
| Education level | Below high school | 4536 (89.1) | 4123 (88.2) | 1.91 | 0.17 | 0.01 |  |
|  | High school or above | 555 (10.9) | 551 (11.8) |  |  |  |  |
| Marital status | Married | 2367 (46.5) | 3242 (85.0) | 35.03 | *p*≤0.001 | 0.05 |  |
|  | Separated, divorced and widowed | 510 (10.0) | 537 (14.1) |  |  |  |  |
|  | Never married | 35 (0.7) | 36 0.9 () |  |  |  |  |
| **Health and behavior variables at 2013** |  |  |  |  |  |  |  |
| BMI *^%^* |  | 24.09 (3.93) | 23.53 (3.86) | 5.83 | *p*≤0.001 | 0.04 |  |
| Smoking status | No | 3557 (69.9) | 3329 (71.2) | 2.20 | 0.14 | 0.01 |  |
|  | Yes | 1534 (30.1) | 1344 (28.8) |  |  |  |  |
| Alcohol status | No | 3381 (66.4) | 3228 (69.0) | 7.82 | *p*≤0.001 | 0.03 |  |
|  | Yes | 1711 (33.6) | 1447 (31.0) |  |  |  |  |
| **Current medical conditions at 2013** |  |  |  |  |  |  |  |
| Anti-dyslipidemia | No | 4732 (92.9) | 4425 (94.7) | 12.35 | *p*≤0.001 | 0.04 |  |
|  | Yes | 360 (7.1) | 250 (5.3) |  |  |  |  |
| Anti-hypertension | No | 3891 (76.4) | 3776 (80.8) | 27.40 | *p*≤0.001 | 0.04 |  |
|  | Yes | 1201 (23.6) | 899 (19.2) |  |  |  |  |
| Anti-diabetes | No | 4813 (94.5) | 4451 (95.2) | 2.36 | 0.12 | 0.00 |  |
|  | Yes | 279 (5.5) | 224 (4.8) |  |  |  |  |
| **Preliminary variables at 2013** |  |  |  |  |  |  |  |
| Depression *^%^* |  | 7.85 (5.71) | 8.13 (6.01) | 2.01 | 0.04 | 0.04 |  |
| **Demographic characteristic variables at 2015** |  |  |  |  |  |  |  |
| Age (years) *^%^* |  | **62.79 (8.49)** | **64.75 (10.5)** | **10.08** | ***p*≤0.001** | **0.19** |  |
| Sex | Male | 2367 (46.5) | 2118 (45.3) | 1.31 | 0.24 | 0.01 |  |
|  | Female | 2721 (53.5) | 2555 (54.7) |  |  |  |  |
| Hukou | Agricultural hukou | 4183 (82.2) | 3676 (78.6) | 19.55 | *p*≤0.001 | 0.04 |  |
|  | Non-agricultural hukou | 907 (17.8) | 999 (21.4) |  |  |  |  |
| Education level | Below high school | 4536 (89.1) | 4123 (88.2) | 1.91 | 0.17 | 0.01 |  |
|  | High school or above | 555 (10.9) | 551 (11.8) |  |  |  |  |
| Marital status | Married | **4466 (87.7)** | **2918 (82.4)** | **43.95** | ***p*≤0.001** | **0.05** |  |
|  | Separated, divorced and widowed | **594 (11.7)** | **586 (10.6)** |  |  |  |  |
|  | Never married | **32 (0.6)** | **30 (1.0)** |  |  |  |  |
| **Health and behavior variables at 2015** |  |  |  |  |  |  |  |
| BMI *^%^* |  | 24.07 (4.03) | 23.96 (14.53) | 0.32 | 0.75 | 0.04 |  |
| Smoking status | No | 3706 (72.8) | 2646 (75.1) | 5.67 | 0.02 | 0.03 |  |
|  | Yes | 1384 (27.2) | 877 (24.9) |  |  |  |  |
| Alcohol status | No | 3333 (65.5) | 2479 (70.4) | 22.76 | *p*≤0.001 | 0.04 |  |
|  | Yes | 1758 (34.5) | 1044 (29.6) |  |  |  |  |
| **Current medical conditions at 2015** |  |  |  |  |  |  |  |
| Anti-dyslipidemia | No | 4633 (91.0) | 4399 (94.1) | 33.89 | *p*≤0.001 | 0.04 |  |
|  | Yes | 459 (9.0) | 276 (5.9) |  |  |  |  |
| Anti-hypertension | No | **3734 (73.3)** | **3742 (80.0)** | **61.16** | ***p*≤0.001** | **0.05** |  |
|  | Yes | **1358 (26.7)** | **933 (20.0)** |  |  |  |  |
| Anti-diabetes | No | 4775 (93.8) | 4463 (95.5) | 13.60 | *p*≤0.001 | 0.03 |  |
|  | Yes | 317 (6.2) | 212 (4.5) |  |  |  |  |
| **Preliminary variables at 2015** |  |  |  |  |  |  |  |
| CRP *^%^* |  | 1.93 (1.76) | 1.92 (1.77) | 0.20 | 0.84 | 0.00 |  |
| Depression *^%^* |  | 8.14 (6.36) | 8.45 (6.66) | 3.17 | *p*≤0.001 | 0.04 |  |

Notes. Values are M (SD) =means (standard deviations) for continuous variables with *%* above and percentages (%) for categorical variables. BMI=body-mass index. CRP=C-reactive protein. V in effects sizes=Cramer’s V. *d* in effects sizes=Cohen’s *d*. Comparison and effect size were reported in absolute values. Significant differences were written in bold.
